# Sphingosine Kinases/Sphingosine-1-Phosphate and Death Signalling in APP-Transfected Cells

**DOI:** 10.1007/s11064-014-1240-3

**Published:** 2014-01-23

**Authors:** Magdalena Gassowska, Magdalena Cieslik, Anna Wilkaniec, Joanna B. Strosznajder

**Affiliations:** Department of Cellular Signalling, Mossakowski Medical Research Centre, Polish Academy of Sciences, Pawinskiego 5, 02-106 Warsaw, Poland

**Keywords:** Alzheimer’s disease, Amyloid β-peptides, Sphingolipids, Sphingosine kinases, Sphingosine-1-phosphate, Dopaminergic cells

## Abstract

It has been postulated that disturbances in the sphingolipid metabolism play a key role in the pathogenesis of Alzheimer’s disease (AD). An alteration in sphingosine kinases 1, 2 (SphK1/2) and sphingosine-1-phosphate (S1P) was recently reported in AD. However, the effect of AD-related amyloid beta (Aβ) peptides on SphK1/2 and the role of S1P in Aβ toxicity have not been fully elucidated. In this study the relationship between the Aβ concentration and SphK1/2 expression/activity was analysed in PC12 cells transfected with the Aβ precursor protein, wild-type (APP_wt_) or bearing a double Swedish mutation (APP_sw_). The role of SphK(s)/S1P in cell survival and death was also investigated. Our results indicated that endogenously liberated Aβ significantly decreases expression and activity of SphK1/2. The SphK(s) inhibitor (SKI II, 10 μM) decreased the viability of APP_wt_, APP_sw_ as well as empty vector-transfected PC12 control cells. Our data demonstrated that expression of S1P receptor-1 (S1P1) was significantly reduced in APP-transfected cells. The effect of S1P applied exogenously was cell type-dependent. In control and APP_wt_ cells S1P reduced the effect of the SphK1 inhibitor on death signalling. Conversely, it decreased the survival of APP_sw_ cells and had no protective effect on cells treated with SKI II. Using the S1P1 agonist (SEW2871, 5 μM) and antagonist (W123, 20 μM), we demonstrated that the cytoprotective effect of S1P was receptor-independent. Summarising, we showed that Aβ peptides evoke down-regulation of gene expression and activity for SphK(s) and S1P1. Inhibition of SphK(s) significantly decreased cell survival. The effect of exogenous S1P depended on the concentration of Aβ peptides.

## Introduction

Sphingosine kinases 1 (SphK1) and 2 (SphK2) are conserved lipid kinases which regulate diverse biological processes in neurons. SphK1 and SphK2 phosphorylate sphingosine to sphingosine 1-phosphate (S1P), which is a bioactive lipid mediator [1–3]. Depending on the concentration and intracellular localisation, S1P plays an important role in the regulation of cell proliferation, differentiation, and motility, and is also involved in a wide array of stress signals, which leads to growth arrest and apoptosis [4–6]. Extracellular S1P is an agonist of 5 surface G-protein-coupled receptors (GPCR), termed S1P1-5, but it may also act intracellularly as a second messenger and directly influence different cellular targets [7, 8]. In addition to mobilising intracellular Ca^2+^ [9], S1P can regulate histone deacetylases and the ubiquitin ligase activity of tumour necrosis factor receptor-associated factor 2 [10–12]. Stimulation of the S1P1–S1P3 receptors may activate (PI3K)/AKT, phospholipase C (PLC), extracellular signal regulated kinases 1/2 (ERK1/2) or GTPases [13–15]. There have been studies which suggest that the S1P signalling response may be opposite, depending on through which kinase(s) it is generated. While SphK1 activity regulates cell growth and proliferation and exerts anti-apoptotic effects [16], the action of SphK2 is implicated in proapoptotic processes [17, 18]. Moreover, SphK1-dependent signalling involves inhibition of the ceramide synthases and activation of the endoplasmic reticulum S1P lyase, which is responsible for the degradation of S1P. Conversely, SphK2 activity promotes the dephosphorylation of S1P to sphingosine, which may either be re-phosphorylated or converted to ceramide [1, 18, 19]. Thus, the differential effects of the two SphK(s) may in part be due to their opposite roles in the regulation of ceramide levels.

The perturbed sphingolipid metabolism is a fundamental event in the neurodegeneration that occurs in Alzheimer’s disease (AD) and other neurological disorders, including brain ischemia, hypoxia and inflammation [1]. AD, a progressive neurodegenerative disorder, is the most severe form of dementia. One important pathologic feature of AD is the formation of brain extracellular senile plaques, whose major components are beta-amyloid (Aβ) peptides which derive from the Aβ precursor protein’s (APP) sequential cleavage by beta- and gamma-secretases [20, 21]. APP and the two secretases are integral membrane proteins that are located in the lipid rafts, which mainly consist of sphingolipids. Thus, lipid composition, biostat and concentration could play an important role in the trafficking of secretases and APP metabolism [22]. It has been shown that Aβ interacts with plasma membrane phospholipids, thus leading to changes in the biophysical properties of the bilayer and causing disturbances in the sphingolipids, glycosphingolipids and cholesterol that may alter the function of lipid rafts [23].

Both an increased level of ceramide and a sphingosine/S1P rheostat imbalance are observed from the earliest clinically recognisable stages of AD [1]. In vivo studies showed a decrease in the concentration of S1P and ceramide accumulation which was proportional to the degree of cognitive impairment, loss of intellectual ability and loss of neurons [2, 23, 24]. Moreover, dramatic changes in other sphingolipid content were also demonstrated, e.g. ceramides and galactoceramides were increased while sulfatides and phospholipids were reduced. Recent studies showed an increased level of ceramide with a simultaneous decrease in sphingomyelin levels in the brain and in the cerebrospinal fluid of AD patients as compared with samples from healthy elderly donors [1, 23, 24]. Increased amounts of sphingosine with concomitant decreased levels of S1P were also found in cytosolic fractions prepared from AD brains [1, 22]. Additionally, in those preparations the profile of sphingolipid metabolising enzymes was shifted towards promotion of ceramide synthesis [1].

It has been indicated that sphingolipids, the fundamental component of lipid rafts, influence the proteolytic cleavage of APP and the production of Aβ. Ceramide as well as S1P produced by SphK2 enhance Aβ generation by posttranslational stabilisation of β-site APP cleaving enzyme-1 (BACE-1). In contrast, sphingomyelin (SM) decreases Aβ production by inhibition of the γ-secretase [2, 23–25]. Aβ may in turn cause deregulation of the sphingolipid metabolism. An in vitro study showed that Aβ_42_ down-regulates SM through activation of neutral sphingomyelinase (nSMase), which leads to an increased level of ceramide, a mediator of Aβ-inducted apoptosis [24, 26]. Under pathological conditions such as AD, cleavage of APP and Aβ generation are probably responsible for deregulated sphingolipid homeostasis [24].

The significance of Aβ in the alteration of sphingosine kinases/S1P and their involvement in the regulation of cell viability are not well understood. The aim of our study was to investigate the relationship between the level of Aβ and SphK1/2 expression/activity and its role in cell survival and death. Moreover, the effect of exogenously added S1P on cell viability was evaluated in an experimental model of AD.

## Materials and Methods

### Chemicals

The following antibodies were used in the current study: anti-SphK1 (Cell Signalling, Beverly, MA, USA), anti-GAPDH, anti-rabbit IgG (Sigma-Aldrich, St. Louis, MO, USA). Reagents for reverse transcription (High Capacity RNA-to-cDNA Master Mix) and PCR (Gene Expression Master Mix) were from Applied Biosystems (Applied Biosystems, Foster City, CA, USA). NBD-Sphingosine was obtained from Avanti Polar Lipids (Alabaster, AL, USA). Cell culture reagents: Dulbecco’s modified Eagle’s medium (DMEM), fetal bovine serum (FBS), horse serum (HS), penicillin, streptomycin, G418, l-glutamine and other reagents such as: deoxyribonuclease I, 3-(4,5-dimethyl-2-tiazolilo)-2,5-diphenyl-2H-tetrazolium bromide (MTT), TRI-reagent, polyethylenoimine (PEI), dimethyl sulfoxide (DMSO) and all other common reagents were from Sigma-Aldrich (St. Louis, MO, USA). Specific SphK(s) inhibitor: 4-[4-(4-chloro-phenyl)-thiazol-2-ylamino]-phenol (SKI II) was obtained from Sigma-Aldrich (St. Louis, MO, USA). *N*,*N*-dimethylsphingosine (DMS) was obtained from Biomol (Plymouth, PA, USA). 2-Amino-4-octadecene-1,3-diol 1-phosphate (S1P) was from Enzo Life Sciences (Lausen, Switzerland). 5-[4-phenyl-5-(trifluoromethyl)-2-thienyl]-3-[3-(trifluoromethyl)phenyl]-1,2,4-oxadiazole (SEW-2871) and 3-(2-(3-hexylphenylamino)-2-oxoethylamino)propanoic acid (W123) were from Cayman Chemical (Ann Arbor, MI, USA).

### Cell Culture

Rat pheochromocytoma (PC12) cells and their derivative clones stably expressing human APP were a kind gift from Dr. W. E. Mueller (University of Frankfurt, Frankfurt am Main, Germany). The studies were carried out using: empty vector-transfected cells (PC12), cells transfected with the human wild-type APP gene (APP_wt_), and cells transfected with the Swedish mutated (K670M/N671L) gene (APP_sw_). All cell lines were cultured in DMEM supplemented with 10 %-heat-inactivated FBS and 5 % heat-inactivated HS, 50 U/ml penicillin/streptomycin, 2 mM l-glutamine and 400 μg/ml G418. Cells were maintained at 37 °C in a humidified incubator in 5 % CO_2_ atmosphere.

### Cell Treatment Protocols

Equal PC12 cell numbers were seeded into 96-well 0.1 % polyethyleneimine-coated plates or 35 mm^2^ dishes and after 24 h the growth medium was changed to a low-serum medium (DMEM supplemented with 2 % FBS, 50 U/ml penicillin/streptomycin and 2 mM l-glutamine). Then the PC12 cells were treated with SphK kinases inhibitor: SKI-II (10 μM), S1P (1 μM), selective agonist of S1P1: SEW-2871 (5 μM), and selective antagonist of S1P1: W123 (20 μM), for 24 h.

### Determination of Cell Survival Using the MTT Test

Cell viability was evaluated by reduction of 2-(4,5-dimethylthiazol-2-yl)-2,5-diphenyltetrazolium bromide (MTT) to formazan. After 24 h of treatment with the tested compounds, MTT (2.5 mg/ml) was added to all of the wells. The cells were incubated at 37 °C for 2 h. Then the medium was removed, the formazan crystals were dissolved in DMSO and absorbance at 595 nm was measured.

### Quantitative Real-Time PCR

Reverse transcription was performed using a High Capacity cDNA Reverse Transcription Kit according to the manufacturer’s protocol (Applied Biosystems, Foster City, CA, USA). The level of mRNA for selected genes was analysed using TaqMan Gene Expression Assays (Applied Biosystems, Foster City, CA, USA) according to the manufacturer’s instructions. *Actb* was selected and used in all studies as a reference gene. Plates were analysed on an ABI PRISM 7500 apparatus. The relative level of mRNA was calculated using the ΔΔCt method.

### Western Blot Analysis

Cells were washed three times with ice-cold PBS and lyzed. Protein levels were determined using the Lowry method, and then the samples were mixed with Laemmli buffer and denatured at 95 °C for 5 min. After standard 10 % SDS-PAGE separation, the proteins were transferred to a PVDF membrane and used for immunochemical detection. The membranes were washed for 5 min in TBS-T buffer (100 mM Tris–buffered saline, 140 mM NaCl and 0,1 % Tween 20) (pH 7.6) and the nonspecific bindings were blocked for 60 min at RT in a 5 % non-fat milk solution in TBS-T buffer. After blocking, the membranes were incubated with the primary antibody (rabbit polyclonal anti-SphK1 antibody*)* used at a dilution of 1:250 in TBS-T buffer, overnight at 4 °C. The membranes were then washed three times (5 min each) in TBS-T buffer and incubated for 60 min at RT with a secondary antibody (anti-rabbit antibody IgG) at a dilution of 1:4,000 in a 5 % non-fat milk/TBS-T solution. Then after four washing steps (3× 5 min in TBS-T buffer, 1× 5 min in TBS buffer) antibodies were detected by a chemiluminescent reaction (ECL reagent) (Amersham Biosciences) under standard conditions. After stripping, the immunolabeling of GAPDH was performed on membranes as a loading control in standard conditions.

### Determination of SphK(s) Activity

Sphingosine kinases activity assay was performed according to a previous report [17]. After 24 h incubation, cells were washed with iced PBS and lysed by freeze–thaw cycle in 50 mM HEPES (10 mM KCl, 15 mM MgCl_2_, 0.1 % Triton X-100, 20 % glycerol, 2 mM orthovanadate, 2 mM dithiothreitol, 10 mM NaF, 1 mM deoxypyridoxine, and EDTA-free complete protease inhibitor) (pH 7.4) (Roche Applied Science). Lysates were cleared by centrifugation at 15.000 rpm for 5 min. The lysates and NBD-Sphingosine (10 μM final) (Avanti Polar Lipids) were mixed in the reaction buffer (50 mM HEPES, 15 mM MgCl_2_ and 0.5 mM KCl, 10 % glycerol and 2 mM ATP) (pH 7.4) and incubated for 30 min at 30 °C. The reactions were stopped by the addition of equal amount of 1 M potassium phosphate (pH 8.5), followed by addition of 2.5-fold chloroform/methanol (2:1), and then centrifuged at 15.000 rpm for 1 min. Only the reactant NBD-S1P, but not the substrate NBD-Sphingosine, was collected in alkaline aqueous phase. After aqueous phase was combined with an equal amount of dimethylformamide, the fluorescence value was read (λ_ex_ = 485 nm, λ_em_ = 538 nm) [27].

### Statistical Analysis

The results were expressed as mean values ± SEM. Differences between the means were analysed using a Student’s *t* test for two groups or one-way analysis of variance ANOVA with Bonferroni’s post hoc test among multiple groups. Statistical significance was accepted at *p* < 0.05. The statistical analyses were performed using Graph Pad Prism version 4.0 (Graph Pad Software, San Diego, CA, USA).

## Results

**Fig. 1:**
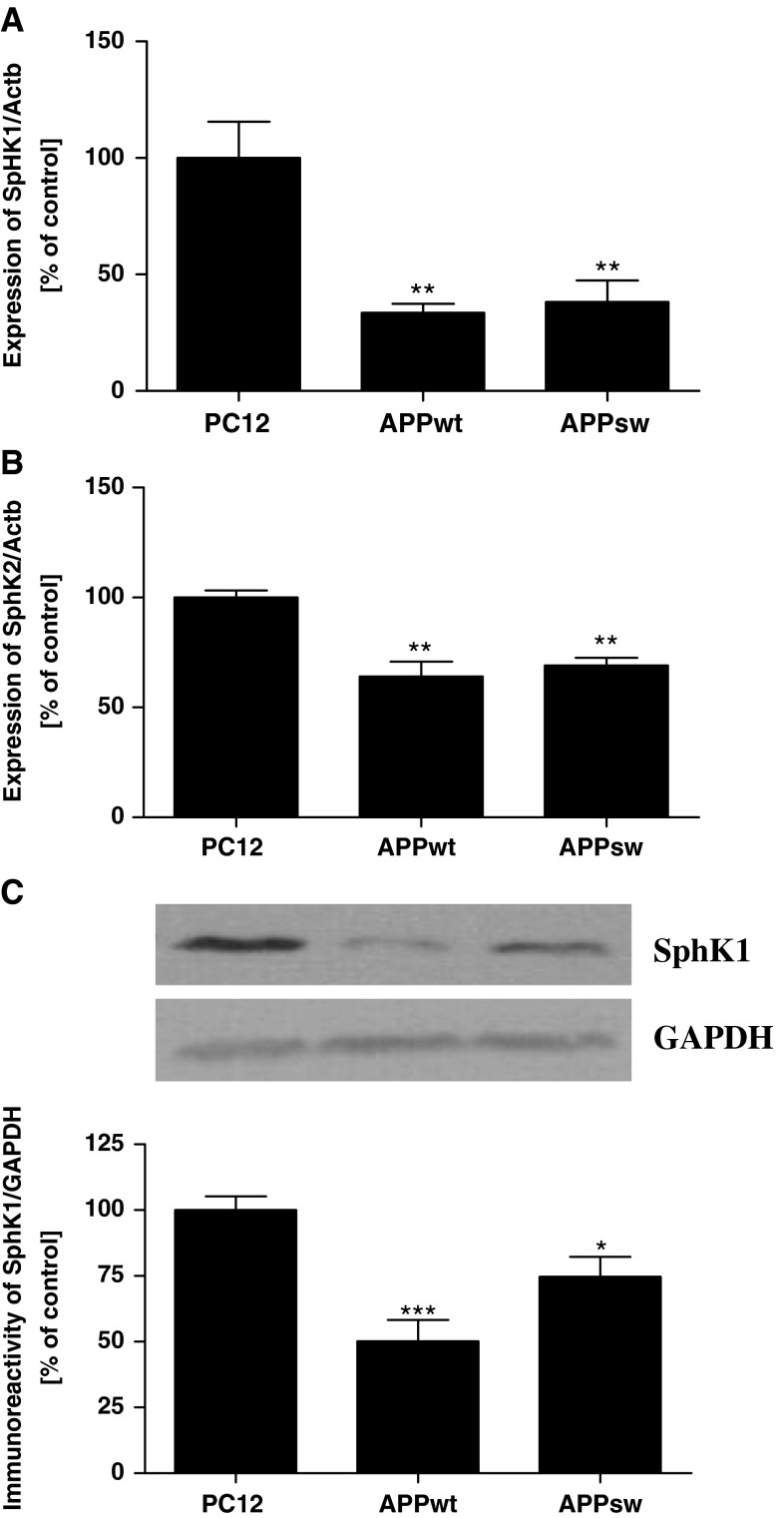
Level of mRNA for SphK1 and SphK2 and protein level of SphK1 in APP-transfected cells. Gene expression for SphK1 (**a**) and SphK2 (**b**) was measured with real-time PCR. (**c**) Total level of SphK1 normalized by GAPDH was determined using Western blot analysis. Data represent the mean value ± SEM for six independent experiments carried out in triplicate. **p < 0.01 versus PC12 cells (cells transfected with an empty vector); ***p < 0.001 versus PC12 cells; *p < 0.05 versus PC12 cells, using one-way ANOVA followed by Bonferroni’s Multiple Comparison Test

**Fig. 2:**
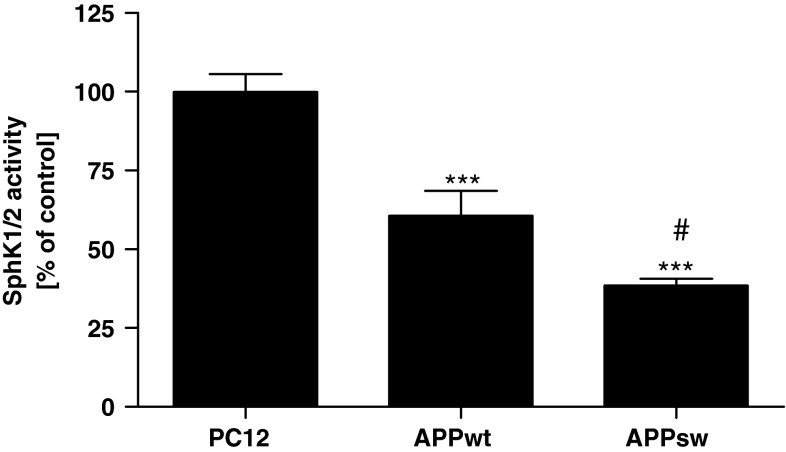
The SphK1/2 activity in APP-transfected cells. The activity of SphK(s) was determined spectrofluorimetically with NBD-Sphingosine as described in “Materials and Methods” section. Data represent the mean value ± SEM for six independent experiments carried out in triplicate. ***p < 0.001 versus PC12 cells; ^#^ *p* < 0.05 versus APP_wt_ cells using a one-way ANOVA followed by Bonferroni’s Multiple Comparison Test

To study the deregulation of the sphingolipid metabolism induced by Aβ peptides, we used PC12 cells stably transfected with wild-type APP (APP_wt_) and APP with the Swedish double mutation (APP_sw_) which secreted, respectively, 2.8 and 4.8 times more Aβ as compared to the PC12 control cells [20, 28]. Ultrastructural analysis revealed that in APP_sw_ overexpressing cells there was formation of an intracellular network of randomly orientated fibrous aggregates of Aβ [29]. By using this in vitro model we observed that endogenously liberated Aβ significantly decreased expression of sphingosine kinase 1 (SphK1, Fig. 1a) and sphingosine kinase 2 (SphK2, Fig. 1b) with concomitant decline in SphK1 protein level (Fig. 1c). This effect, however, did not depend on the concentration of Aβ in cells and was comparable for both APP_wt_ and APP_sw_ cells. As presented in Fig. 1c the immunoreactivity of SphK1 was significantly reduced by about 50 % in APP_wt_ cells and only by about 25 % in APP_sw_ cells. In contrast to changes in gene expression and protein level, the activity of SphK(s) was significantly reduced in APP transfected cells in Aβ concentration dependent manner. The SphK(s) activity was substantially lower in the APP_sw_ cells when compared to the APP_wt_ cells (Fig. 2).

**Fig. 3:**
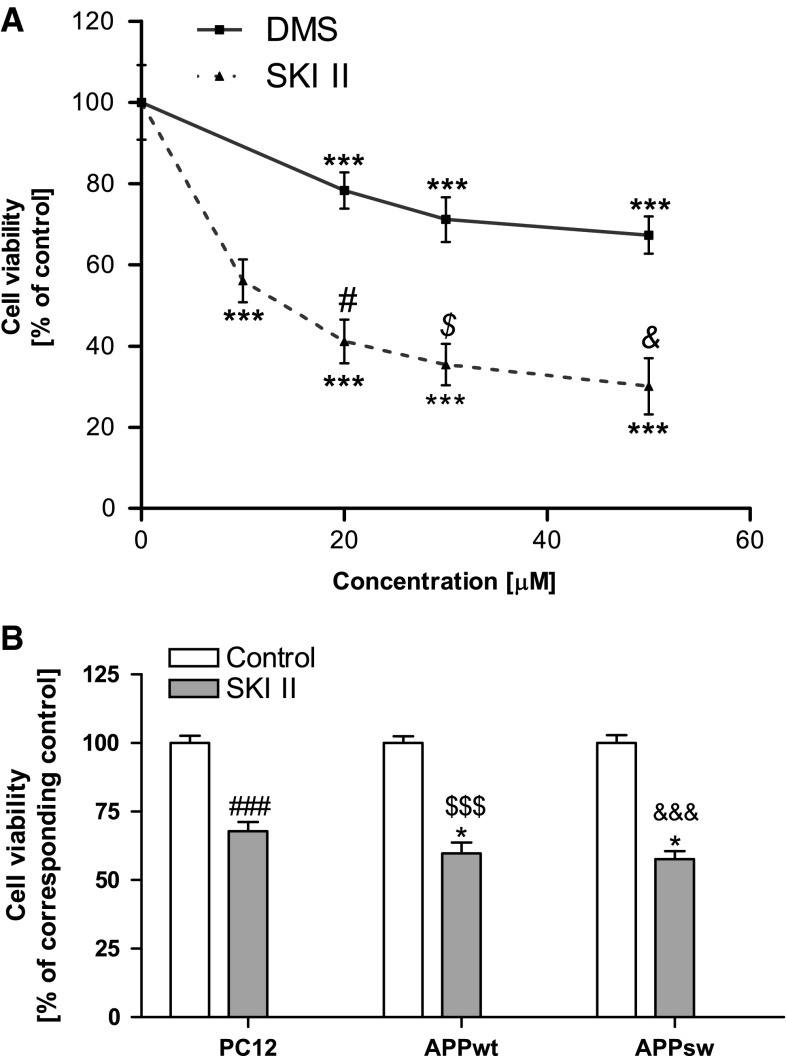
Effect of SphK inhibitors on the viability of PC12 and APP-transfected cells. Cell viability was determined spectrophotometrically using the MTT assay. (**a**) Concentration-dependent effect of SphK(s) inhibitors: DMS and SKI II on the viability of PC12 cells after 24 h incubation. (**b**) Effect of SphK(s) inhibitor: SKI II (10 μM) on APP-transfected cell viability. Data represent the mean value ± SEM for three separate experiments carried out in triplicate. ***p < 0.001 versus control (non-treated) PC12 cells; ^#^ *p* < 0.001 versus 20 µM DMS-treated PC12 cells; ^$^ *p* < 0.001 versus 30 µM DMS-treated PC12 cells; ^&^ *p* < 0.001 versus 50 µM DMS-treated PC12 cells; ^###^ *p* < 0.0001 versus non-treated PC12 cells; ^$$$^ *p* < 0.0001 versus non-treated APP_wt_ cells; ^&&&^ *p* < 0.0001 versus non-treated APP_sw_ cells; *p < 0.05 versus SKI II-treated PC12 cells, using one-way ANOVA followed by Bonferroni’s Multiple Comparison Test

To test whether alteration of SphK(s) may have deleterious consequences, we studied the effect of their inhibition on cell viability. The MTT test revealed that less specific SphK(s) inhibitor (DMS) and a more specific (SKI II) inhibitor of SphK1 reduced PC12 control cell viability in a dose-dependent manner, causing, respectively, the death of 33 and 70 % of cells at a 50 μM concentration (Fig. 3a). Endogenously liberated Aβ did not significantly decrease viability of the APP_wt_ and APP_sw_ cells (MTT test) after 24 h of cultivation (Fig. 3b). The specific SphK(s) inhibitor (SKI II, 10 μM) had a negative effect on the viability both APP-transfected and PC12 control cell line (Fig. 3b).

**Fig. 4:**
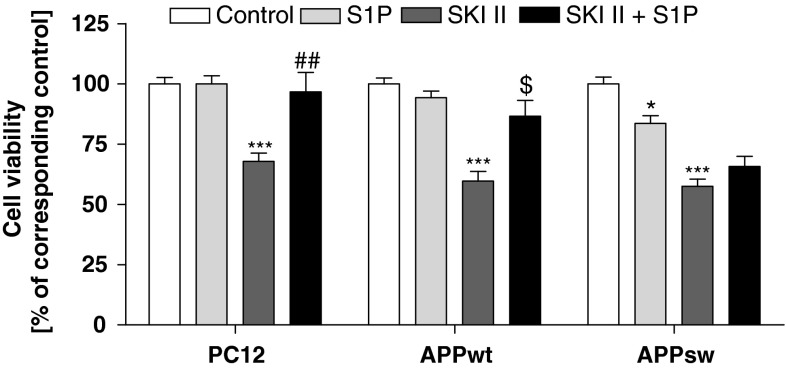
Effect of S1P on SKI II-evoked decrease in cell viability. PC12 and APP-transfected cells were pre-incubated with 1 µM S1P for 5 min, and then 10 µM SKI II was added for 24 h. Cell viability was spectrophotometrically determined using the MTT assay. Data represent the mean value ± SEM for three separate experiments carried out in triplicate. *p < 0.05 versus non-treated APP_sw_ cells; ***p < 0.001 versus corresponding control; ^##^ *p* < 0.01 versus SKI II-treated PC12 cells; ^$^ *p* < 0.05 versus SKI II-treated APP_wt_ cells, using one-way ANOVA followed by Bonferroni’s Multiple Comparison Test

Cell death induced by SKI II may occur due to reduced production of S1P, the main product of enzymatic activity of SphK(s). In this experimental condition the level of sphingosine and subsequently ceramide should increase, thus causing disturbances in the sphingolipid rheostat. In the following experiments the role of S1P on cell viability was investigated. This sphingolipid did not have an effect on the viability of PC12 control and APP_wt_ cells in control conditions (Fig. 4); however, it significantly protected those cells against death induced by SphK(s) inhibition (Fig. 4). Interestingly, S1P caused a decrease of APP_sw_ cell viability and was ineffective when those cells were subjected to SKI II (Fig. 4).

**Fig. 5:**
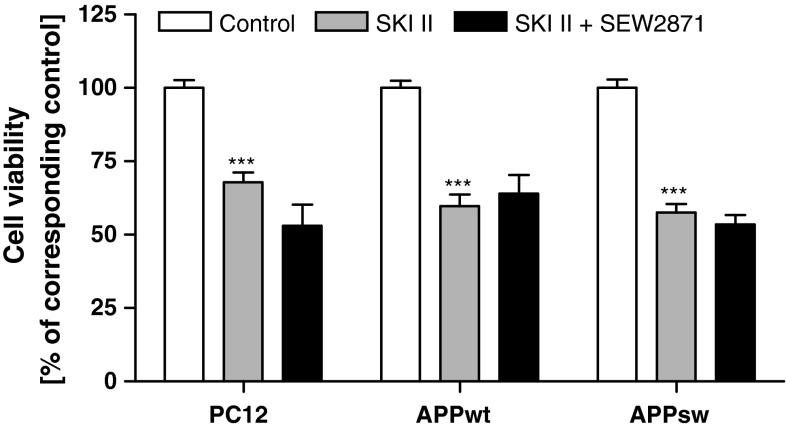
Effect of S1P1 agonist on SKI II-evoked decrease in cell viability. Cells were incubated with 10 µM SKI II in the presence of 5 µM SEW2871 for 24 h. Cell viability was spectrophotometrically determined using the MTT assay. Data represent the mean value ± SEM for three separate experiments carried out in triplicate. ***p < 0.001 versus corresponding control, using one-way ANOVA followed by Bonferroni’s Multiple Comparison Test

Subsequently, we investigated the mechanism of the protective effect of S1P in PC12 control and APP_wt_ cells. To test whether sphingosine 1 phosphate receptor-1 (S1P1) was involved in this phenomenon, we used a specific agonist of S1P1 (SEW2871, 5 μM); however, we did not observe the beneficial effect of this compound on any of the cell lines that were tested (Fig. 5).

**Fig. 6:**
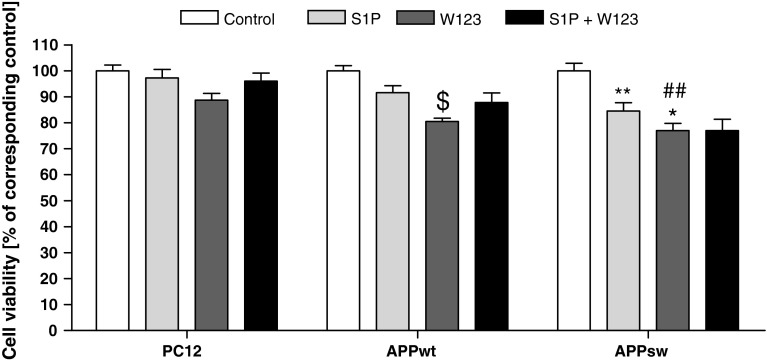
Effect of S1P on S1P1 antagonist-evoked decrease in cell viability. Cells were incubated with 1 µM S1P in the presence of 20 µM W123 for 24 h. Cell viability was spectrophotometrically determined using the MTT assay. Data represent the mean value ± SEM for three separate experiments carried out in triplicate. ^ $^ *p* < 0.05 versus non-treated APP_wt_ cells; ^##^ *p* < 0.01 versus non-treated APP_sw_ cells; *p < 0.05 versus W123-treated PC12 cells; **p < 0.05 versus non-treated APP_sw_ cells, using one-way ANOVA followed by post hoc Bonferroni’s Multiple Comparison Test

**Fig. 7:**
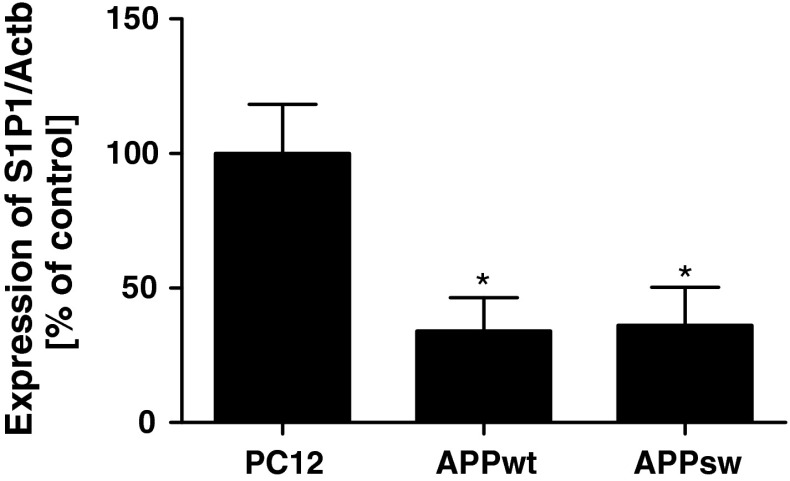
Expression of S1P1 in APP-transfected cells. Gene expression was measured with real-time PCR. Data represent the mean value ± SEM for three independent experiments carried out in triplicate. *p < 0.05 versus PC12 cells, using one-way ANOVA followed by Bonferroni’s Multiple Comparison Test

Additionally, the selective antagonist of S1P1 (W123, 20 μM) induced cell death exclusively in APP-transfected cells, and exogenously added S1P did not have any effect on this negative action (Fig. 6). Moreover, expression of S1P receptor-1 was significantly decreased in both APP_wt_ and APP_sw_ cells (Fig. 7), which may explain the observed ineffectiveness of SEW2871 and suggest that the protective effect of S1P is not mediated by S1P1.

## Discussion

Recent data demonstrated that sphingolipid metabolism alteration may play a crucial role in the ethiopathology of AD. An increased amount of sphingosine and a decreased amount of S1P were found in the cytosolic fraction prepared from the autopsies of AD brains [2], thus suggesting that the activity of S1P synthesising/degrading enzymes may be greatly disturbed in the course of dementia.

A major finding of the present study is that endogenously liberated Aβ peptides caused a significant decrease in the expression of SphK1 and 2 in APP-transfected PC12 cell lines. These data correspond to previous findings which indicated that exposure to Aβ_25–35_, the neurotoxic fragment of the Aβ peptide, induced strong inhibition of SphK1 in neuronal SHSY5Y cells. However, in those experimental conditions Aβ_25–35_ did not significantly alter the SphK2. The data of Gomez-Brouchet et al. [30] demonstrated that knock down of SphK1 expression in SHSY5Y cells resulted in activation of the intrinsic pathway of apoptosis. This involved enhanced Bax oligomerisation, mitochondrial membrane permeabilisation, cytochrome c release and caspase activation [30]. Interestingly, these marked changes in the SphK(s) expression level were not correlated with an alteration of cell viability in our APP-transfected cells. These data indicated that simultaneously of cytoprotective events may occur in these cells.

SphK(s) are largely cytosolic enzymes that have been postulated to migrate to membranes where they can phosphorylate sphingosine to bioactive sphingolipid S1P [31–34]. However, a high concentration of SphK1 was also detected in the nucleus. Transient or stable overexpression of SphK1 in NIH 3T3 fibroblasts or HEK293 cells, with a concomitant increased level of S1P, protected against apoptosis which was induced by serum deprivation or ceramide elevation. Endogenous S1P has been reported to regulate intracellular Ca^2+^ mobilisation and to promote cell growth and survival [31, 35]. However, excessive accumulation of S1P may also have a neurotoxic effect, thus causing disruption of ER calcium homeostasis [13].

S1P is short-lived, so the synthetic process catalysed by SphK may be important in maintaining the compound’s cellular levels. Our data show that S1P supplementation exerted a protective effect on PC12 and APP_wt_ cell viability which was significantly decreased by SphK1 inhibition. However, in the APP_sw_ cells, which were characterised by 4.8 times more Aβ secretion as compared to the PC12 control [20, 28], this effect was not observed. Moreover, S1P decreased the viability of the APP_sw_ cells. A study by Takasugi et al. [17] reported up-regulation of SphK2 activity in AD brains and a positive correlation between SphK2 activity and APP processing. SphK2 activity promotes S1P formation, which may activate the β-site APP cleaving enzyme-1 (BACE-1), thus leading to higher liberation of Aβ peptides [17]. S1P was shown to specifically bind to full-length BACE-1 and to increase its proteolytic activity; then this pool of S1P is dephosphorylated to sphingosine and may be either re-phosphorylated by SphK2 or converted to ceramide [1, 17–19]. Thus, the addition of extracellular S1P in APP_sw_ cells may result in an elevation of Aβ and ceramide levels which is responsible for cell death. Pharmacological or genetic inhibition of SphK2 in APP transgenic mice significantly reduced Aβ levels, thus further suggesting the direct involvement of SphK2/S1P in Aβ generation in vivo [17, 21]. The influence of S1P in Aβ release may also be proved by our data, which show that S1P itself had a deleterious effect in APP_sw_ cells. This suggests that excessive Aβ release may be responsible for disturbance of the SphK(s) and ceramide/S1P biostat, which could be a regulator of cell survival and death in our experimental conditions. In line with this hypothesis, it was demonstrated that transient or stable overexpression of SphK1 in NIH 3T3 fibroblasts or HEK293 cells protected against apoptosis induced by serum deprivation or ceramide elevation [36]. Similar data were obtained by Edsall et al. [16], showing that overexpression of SphK1 promotes cell survival in PC12 cells exposed to C_2_-ceramide by increasing the intracellular S1P level with a concomitant reduction of both ceramide and sphingosine production [16]. Moreover, SphK1 overexpression prevented apoptosis by blocking activation of caspase-2, -3 and -7 and the stress-activated protein kinase, c-Jun amino terminal kinase (SAPK/JNK) [22]. On the contrary, SphK1 down-regulation was associated with an accumulation of ceramide and, in consequence, with cell death [37–41]. Moreover, S1P itself had a deleterious effect in those cells.

It is well known that S1P may act intracellularly as a second messenger [8, 42] or extracellularly as a ligand for the G protein-coupled receptors S1P1 to S1P5 [43, 44]. A growing body of evidence suggests that SphK(s) and S1P receptors may play an important role in the pathogenesis of AD and other neurodegenerative disorders, such as multiple sclerosis (MS), HIV and dementia. Here we showed that the protective effect of S1P in PC12 control and APP_wt_ cells was independent of sphingosine-1-phosphate receptor-1 (S1P1) activation. The specific agonist of S1P1 (SEW2871) did not exert a beneficial effect. Our data demonstrated that Aβ peptides significantly decreased expression of S1P1. The experiment of Kaneider et al. [44] demonstrated Aβ_39–43_-dependent regulation of SphK(s) activity and S1P receptor expression in human monocytes. Induction of S1P2 and S1P5 mRNA in Aβ_39–43_ treated cells was indicated [44].

Summarising, we observed decreased expression and activity of SphK1/2 as well as of the expression of S1P1 receptor in APP-transfected cells secreting a different amount of Aβ peptides. Moreover, our data demonstrated that the cytoprotective effect of S1P against death evoked by SphK(s) inhibition was not mediated by S1P1 and depended on the type of cells and the Aβ concentration. The capability of Aβ to modulate the sphingolipid metabolism suggests that this deregulation may be a crucial event that occurs in the course of degeneration.
